# A Cell Cycle-Regulated *Toxoplasma* Deubiquitinase, TgOTUD3A, Targets Polyubiquitins with Specific Lysine Linkages

**DOI:** 10.1128/mSphere.00085-16

**Published:** 2016-06-22

**Authors:** Animesh Dhara, Anthony P. Sinai

**Affiliations:** Department of Microbiology, Immunology, and Molecular Genetics, University of Kentucky College of Medicine, Lexington, Kentucky, USA; Indiana University School of Medicine

**Keywords:** *Toxoplasma gondii*, deubiquitinase, OTU, polyubiquitin, cell cycle

## Abstract

The contribution of ubiquitin-mediated mechanisms in the regulation of the *Toxoplasma gondii* cell cycle has remained largely unexplored. Here, we describe the functional characterization of a *T. gondii* deubiquitinase (TGGT1_258780) of the *o*varian-*tu*mor domain-containing (OTU) family, which, based on its structural homology to the human OTUD3 clade, has been designated TgOTUD3A. The TgOTUD3A protein is expressed in a cell cycle-dependent manner mimicking its mRNA expression, indicating that it is regulated primarily at the transcriptional level. TgOTUD3A, which was found in the cytoplasm at low levels in G_1_ parasites, increased in abundance with the progression of the cell cycle and exhibited partial localization to the developing daughter scaffolds during cytokinesis. Recombinant TgOTUD3A but not a catalytic-site mutant TgOTUD3A (C229A) exhibited activity against poly- but not monoubiquitinated targets. This activity was selective for polyubiquitin chains with preference for specific lysine linkages (K48 > K11 > K63). All three of these polyubiquitin linkage modifications were found to be present in *Toxoplasma*, where they exhibited differential levels and localization patterns in a cell cycle-dependent manner. TgOTUD3A removed ubiquitin from the K48- but not the K63-linked ubiquitinated *T. gondii* proteins independently of the modified target protein, thereby exhibiting the characteristics of an exodeubiquitinase. In addition to cell cycle association, the demonstration of multiple ubiquitin linkages together with the selective deubiquitinase activity of TgOTUD3A reveals an unappreciated level of complexity in the *T. gondii* “ubiquitin code.”

**IMPORTANCE** The role of ubiquitin-mediated processes in the regulation of the apicomplexan cell cycle is beginning to be elucidated. The recent analysis of the *Toxoplasma* “ubiquitome” highlights the importance of ubiquitination in the parasite cell cycle. The machinery regulating the ubiquitin dynamics in *T. gondii* has remained understudied. Here, we provide a biochemical characterization of an OTU (*o*varian *tu*mor) family deubiquitinase, TgOTUD3A, defining its localization and dynamic expression pattern at various stages of the cell cycle. We further establish that TgOTUD3A has activity preference for polyubiquitin chains with certain lysine linkages—such unique activity has not been previously reported in any apicomplexan. This is particularly important given the finding in this study that *Toxoplasma gondii* proteins are modified by diverse lysine-linked polyubiquitin chains and that these modifications are very dynamic across the cell cycle, pointing toward the sophistication of the “ubiquitin code” as a potential mechanism to regulate parasite biology.

## Abstract (precis)

The role of ubiquitin-mediated processes in the regulation of the apicomplexan cell cycle is beginning to be elucidated. The recent analysis of the *Toxoplasma* “ubiquitome” highlights the importance of ubiquitination in the parasite cell cycle. The machinery regulating the ubiquitin dynamics in *T. gondii* has remained understudied. Here, we provide a biochemical characterization of an OTU (*o*varian *tu*mor) family deubiquitinase, TgOTUD3A, defining its localization and dynamic expression pattern at various stages of the cell cycle. We further establish that TgOTUD3A has activity preference for polyubiquitin chains with certain lysine linkages—such unique activity has not been previously reported in any apicomplexan. This is particularly important given the finding in this study that *Toxoplasma gondii* proteins are modified by diverse lysine-linked polyubiquitin chains and that these modifications are very dynamic across the cell cycle, pointing toward the sophistication of the “ubiquitin code” as a potential mechanism to regulate parasite biology.

## INTRODUCTION

*Toxoplasma gondii*, a protozoan parasite of veterinary and human importance, causes toxoplasmosis, which presents as symptomatic disease in immunocompromised humans or animals and can be vertically transmitted during pregnancy (1, 2). About a third of the human population worldwide is infected by this organism, where the control of acute infection results in the establishment of a latent encysted form within the host that is capable of reactivation in the context of immune suppression (3, 4). At the heart of the clinical manifestations of these diseases is the capacity to replicate within the host, resulting in the destruction of infected cells and cycles of reinfection (5). *T. gondii* replicates in a unique mechanism called endodyogeny, in which two daughter buds are formed within the mother during cytokinesis (5, 6). While elements of the mother parasite, including genome-containing organelles (nucleus, mitochondrion, and apicoplast), are duplicated and inherited, other maternal components are degraded and formed *de novo* in the daughters (5, 7). The degradation of maternal components provides the building blocks of developing daughter parasites and must be tightly regulated. Both selective ubiquitin (Ub)-mediated turnover and bulk turnover by autophagy are likely involved in maternal degradation (8). Regardless of the mechanism underlying maternal degradation, the process has to be selective for maternal components while avoiding the turnover of the daughters to ensure their survival. Such regulation can be facilitated by selective marking of maternal components for degradation and/or the spatial segregation of the maternal and daughter components by selective access to the degradative machinery. Such functional control is further necessitated as demonstrated by the fact that uncontrolled activation of autophagy leads to the programmed cell death pathway in *Toxoplasma* (9).

In eukaryotes, posttranslational modification by conjugating monomeric ubiquitin or polyubiquitin (poly-Ub) to a protein serves as a signal for selective degradation by the proteasome (10). Ubiquitination may also have roles in signaling and directing vesicular traffic (11–13). Using a cross-reacting human monoclonal antibody (MAb) against the 20S proteasome, Paugam et al. (14) first demonstrated the existence of the proteasome in *T. gondii*. Subsequent genome-wide bioinformatic analyses of apicomplexans, including *Toxoplasma*, have identified candidate genes encoding major players of the ubiquitin proteasome system, which includes ubiquitin, ubiquitin-like peptides, ubiquitin modifiers, ubiquitin ligases needed for ubiquitin activation/conjugation/ligation reactions, and deubiquitinases (DUBs) (15). Together, these data point directly to an important role for ubiquitin and ubiquitin-mediated processes in *Toxoplasma*. These bioinformatics findings have been recently confirmed by the application of proteomic approaches to catalog the “ubiquitome” of *T. gondii* (16). This study has not only established that *T. gondii* encodes the capacity to ubiquitinate a significant proportion of its proteome but further demonstrated that many target proteins which get ubiquitinated are also transcriptionally regulated in a cell cycle-dependent manner (16–18). While a few studies have looked into the ubiquitin ligases in Apicomplexa, investigations on DUBs have been limited (15, 19, 20).

The *T. gondii* genome contains about 40 different DUBs, which can be categorized into five different classes (USPs, UCHs, OTUs, Josephins, and metalloproteases) based on the classification of human DUBs (15, 21). We were specifically interested in the DUBs impacting the cell cycle and reasoned that DUBs exhibiting cell cycle-dependent expression had a high likelihood of being involved in cell cycle-related regulation and processes.

Examination of the genome at ToxoDB (http://www.toxodb.org) for cell cycle-regulated expression (17) reveals that mRNA expression of one member of the OTU family DUBs (TGGT1_258780) drops dramatically (8-fold) in parasites transitioning from mitosis to the completion of cytokinesis (17). We have designated this gene *T. gondii* OTUD3A (TgOTUD3A) as described below. Quite significantly, the *Plasmodium falciparum* ortholog (PF3D7_0923100) also has a cell cycle stage-specific transcript expression profile (22), suggesting a potential functional conservation of this OTU DUB across apicomplexan species.

In this study, we characterized the expression and biochemical activity profile of TgOTUD3A against both synthetic substrates and parasite proteins. A detailed functional characterization of TgOTUD3A reveals substrate specificity for different ubiquitin linkages which were found to be present in *Toxoplasma*. This highlights a previously unappreciated level of complexity of the “ubiquitin code” in *Toxoplasma* that suggests that a considerable degree of sophistication for ubiquitin-mediated protein modification exists in the parasite.

## RESULTS

### The *T. gondii* genome encodes several putative OTU cysteine proteases.

The OTUs are the members of a cysteine protease family of deubiquitinases, first identified in *Drosophila melanogaster*, in which the misregulation of the founding member caused ovarian tumors (23). OTU family deubiquitinases have also been found to be important in mammals, where dysregulation is linked with defects in the cell cycle and is associated with diverse cancers (24, 25). We focused on the OTU class of deubiquitinases in *T. gondii* as a part of a broader aim to understand their role in ubiquitin dynamics during the parasite cell cycle.

**FIG 1:**
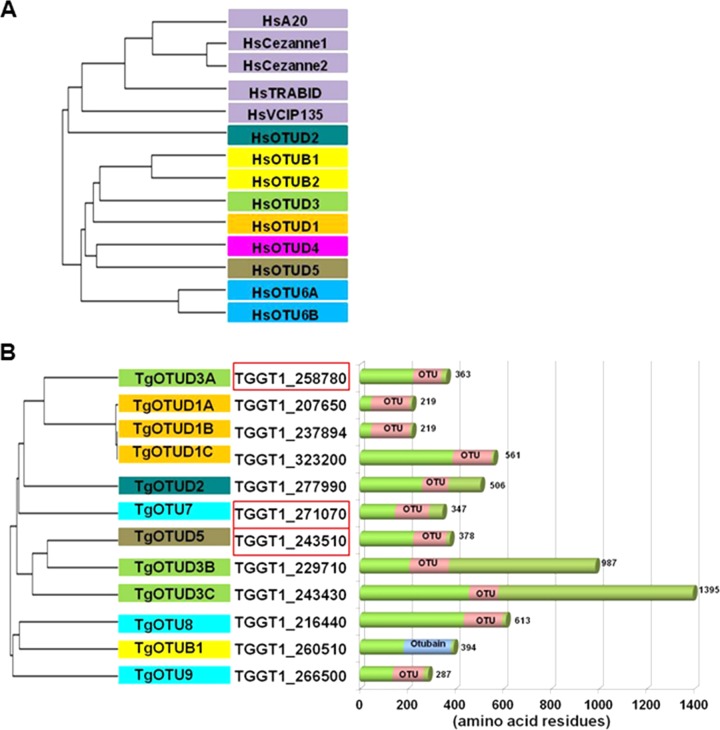
Phylogenetic analysis of human and putative *Toxoplasma* OTUs. (A) Phylogenetic tree of human (Hs) OTUs based on the sequence homology of their OTU domains. Human OTU domain sequences which were used to define this relationship have been described in the work of Mevissen et al. (26). The members of the same OTU clade are marked with the same color. The sequence of and structural information on human OTU domains were used to classify the *Toxoplasma* OTUs (TgOTUs). (B) Phylogenetic tree of putative *Toxoplasma* OTU domains based on the homology of amino acid sequences of the OTU domains. TgOTUs were designated on the basis of SMART alignment to the closest human OTU domains (color codes indicate predicted structural similarity with HsOTU domains). Homologs are designated TgOTU-clade-A/B/C to indicate the members with similar OTU domain structures. The predicted structures of 3 *Toxoplasma* OTUs do not match any human OTU domains, and these have been designated TgOTU7, -8, and -9. The protein length (number of amino acids) and the predicted locations of OTU domains within the individual TgOTU genes are schematically represented. The sequence accession numbers of three OTUs that show cell cycle-associated transcript expression (17) have been marked with a red box. Nucleotide sequence accession numbers for *Toxoplasma* genes were obtained from ToxoDB.

A search for OTU cysteine proteases in the *Toxoplasma* genome database (http://www.toxodb.org) using motif and text searches (see Materials and Methods) revealed 15 putative hits, including two genes (TGGT1_271070 and TGGT1_266500) annotated as hypothetical proteins and one (TGGT1_260510) annotated as a ubiquitin thioesterase. In order to further characterize and classify the putative hits, we performed a BLAST search of primary amino acid sequences of TgOTUs against the sequence and structural information of human OTUs in the Protein Data Bank (http://www.rcsb.org/pdb). Phylogenetic analysis (Fig. 1A) distributes the human OTUs into four distinct clades (OTUB/Otubain, OTUD, A20-like, and OTULIN clades) based on the homology of the OTU domain (26). We classified and named the TgOTUs based on structure prediction analysis (see Materials and Methods) with reference to the human OTU domain-encoding genes (26). The TgOTUs have the highest structural homology to two human ortholog clades (OTUD and OTUB), with neither the human A20-like proteins nor OTULIN subfamily members being represented (Fig. 1B; Table 1) (26). Out of 15 hits in the *T. gondii* genome, we identified the OTU domain in 12 protein sequences. Most of the TgOTUs possess a C-terminal OTU domain (Pfam accession number PF02338) (27), except for TGGT1_243430 and TGGT1_229710, which have the OTU domains in the middle of the protein sequence (Fig. 1B). These two TgOTUs are also very large (1,395 amino acids [aa] and 987 aa, respectively) compared to other TgOTUs (in the range of 200 to 600 aa) (Fig. 1B). Of note, three TgOTUs (TgOTU7 [TGGT1_271070], TgOTU8 [TGGT1_216440], and TgOTU9 [TGGT1_266500]) possess predicted structures that did not match with any of the known human OTU domain clades and so were designated by numbers only. Three genes (TGGT1_410520, TGGT1_362240, and TGGT1_356210) were previously annotated as putative TgOTUs in ToxoDB based on their homology to different ortholog groups, but none of them have true OTU domains and they were therefore excluded from further analyses.

**TABLE 1:** Catalog of *Toxoplasma* OTUs based on amino acid sequence homology and predicted structural similarity relative to characterized human OTUs^a^

TgOTU	Gene identifier	Closest human ortholog	% identity (similarity) to HsOTU domain	Predicted linkage specificity based on HsOTU activity
TgOTUD1A	TGGT1_207650	HsOTUD1	23 (39)	K63
TgOTUD1B	TGGT1_237894	HsOTUD1	23 (37)	K63
TgOTUD1C	TGGT1_323200	HsOTUD1	23 (37)	K63
TgOTUD2	TGGT1_277990	HsOTUD2	43 (63)	K27, K29, K33
**TgOTUD3A**	**TGGT1_258780**	**HsOTUD3**	**28 (46)**	**K6, K11**
TgOTUD3B	TGGT1_229710	HsOTUD3	23 (40)	K6, K11
TgOTUD3C	TGGT1_243430	HsOTUD3	27 (48)	K6, K11
TgOTUD5	TGGT1_243510	HsOTUD5	21 (40)	K48, K63
TgOTUD7	TGGT1_271070	ND		ND
TgOTUD8	TGGT1_216440	ND		ND
TgOTUD9	TGGT1_266500	ND		ND
TgOTUB1	TGGT1_260510	Otubain (HsOTUB1)	27 (49)	K48

a Predicted substrate specificities of TgOTUs for poly-Ub linkage are based solely on the primary sequence data and their relatedness to the human HsOTUs. These may not reflect true linkage specificities determined by biochemical characterization. Such a functional deviation is evident for TgOTUD3A (in bold). Some TgOTU domains lack sequence-based structural homology to human HsOTUs, noted as ND (not determined). Predicted linkage specificity is based on HsOTU activity as reported by Mevissen et al. (26).

### TgOTUD3A expression is variable in the population but uniform within a vacuole.

**FIG 2:**
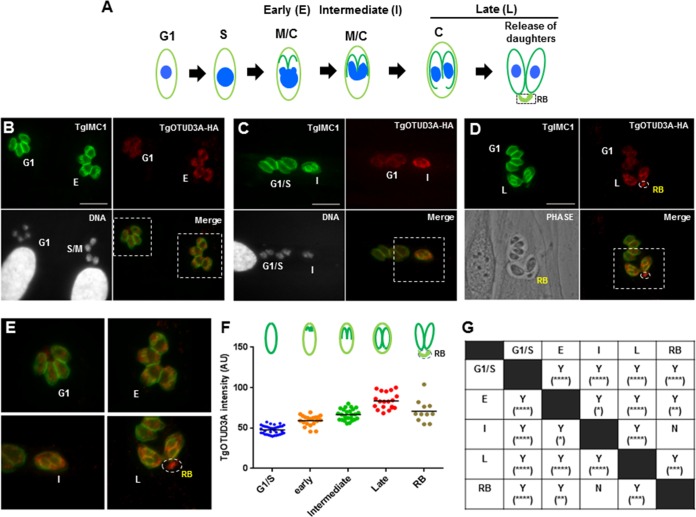
TgOTUD3A expression and localization during endodyogeny. (A) Schematic representation of *T. gondii* endodyogeny: presence of overlapping cell cycle phases. Patterns defining nuclear size, intensity of DNA labeling, and nuclear morphology establish the G_1_, S, and M phases. The staining pattern of TgIMC1 was used as a guide for cytokinesis. The extent of TgIMC1 labeling of daughter scaffolds defines the early (E), intermediate (I), and late (L) stages of cytokinesis. The maternal remnant, the residual body (RB), is indicated. (B) Expression dynamics and localization of TgOTUD3A in a TgOTUD3A-HA-tagged clonal line. Expression of *in situ*-tagged TgOTUD3A-HA is uniform within a vacuole, but intensity differs in adjacent vacuoles, suggesting cell cycle-dependent expression similar to the transcript expression profile. The parasites in G_1_ (left) have lower expression levels than do the ones with early small daughter TgIMC1 scaffolds (E, right). The parasites with early daughter buds (E) transitioning from S phase to mitosis (S/M) are characterized by larger, more intensely labeled nuclei (DNA). (C) The parasite on the right is in the intermediate (I) stage of cytokinesis (based on the length of daughter TgIMC1 scaffolds) and expresses relatively more TgOTUD3A-HA than do ones in G_1_/S (four parasites on the left). (D) Parasites at the late stages of cytokinesis (L; with mature daughter buds) exhibit a higher TgOTUD3A-HA signal than do the ones in G_1_. TgOTUD3A-HA signal is also evident within the residual body (RB). Bars, 10 µm. (E) Enlarged merged images of four stages (G_1_, early, intermediate, and late, highlighted with the dashed boxes in the other panels) show granular, punctate, and cytoplasmic TgOTUD3A-HA signal while showing partial colocalization with the early, intermediate, and late daughter buds. There is no accumulation of TgOTUD3A-HA in the nucleus. The level of TgOTUD3A-HA appears to be highest in the parasites with mature daughter buds (L). The residual body exhibits TgOTUD3A labeling without any significant TgIMC1 signal. (F) Images of HA/TgIMC1 double-labeled TgOTUD3A-HA parasites were acquired at random at a fixed exposure. The HA signal intensity within whole individual parasites was measured in arbitrary units (AU) using ImageJ software and plotted to show the distribution and range of signal intensity at different cell cycle stages based on the TgIMC1 staining pattern (as shown in the cartoon above). For the residual body category, only the signal intensity of the residual body was measured where it was clearly present in the phase images. (G) One-way ANOVA shows that there is a significant difference in TgOTUD3A-HA expression across different cell cycle stages. One-way ANOVA: *F*_4,107_ = 70.45 (*P* < 0.0001). The comparisons between cell cycle stages were done using Tukey’s pairwise multiple-comparison test (α = 0.05). Where the mean fluorescence intensity differed significantly is indicated by Y (for yes), and the level of significance is indicated by the number of asterisks. The letter N (for no) indicates that no significant difference exists between groups.

*Toxoplasma gondii* tachyzoites (associated with the acute asexual phase) and bradyzoites (associated with the chronic asexual phase) replicate predominantly by endodyogeny, an internal budding mechanism where 2 daughters are formed within the mother (5, 28). The functional organization of endodyogeny deviates from the canonical eukaryotic cell cycle in that the specific stages overlap, as seen most dramatically in the fact that cytokinesis is initiated before the completion of mitosis (29) (Fig. 2A). Inner membrane complex 1 (TgIMC1) intensely labels developing and recently emergent parasites (Fig. 2A), as was previously shown (30, 31). The staining pattern of TgIMC1 has been used as a marker of different stages of cytokinesis. Microarray data available in ToxoDB from a cell cycle study on thymidine-synchronized parasites showed that the transcript expression patterns of TgOTUD3A (TGGT1_258780) exhibited a dramatic cell cycle-regulated profile during endodyogeny (17). TgOTUD3A is significantly upregulated during S phase and the early stages of mitosis, following which expression drops dramatically (about 8-fold) when the cell cycle moves from late mitosis to cytokinesis. Expression levels are at their lowest in early G_1_ and subsequently increase as the parasite enters S phase (17).

In order to examine if the transcriptional profile is accurately reflected at the protein level, we epitope tagged TgOTUD3A *in situ* with a C-terminal 3× hemagglutinin (HA) tag (32). Immunofluorescence analysis using anti-HA antibody indicates that there is a range of expression in different vacuoles (Fig. 2B to D). Consistent with synchronous replication within a vacuole, TgOTUD3A expression within any given vacuole remains uniform (Fig. 2B to D). Costaining the TgOTUD3A-HA parasites with anti-TgIMC1 (green) and anti-HA (red) antibodies (Fig. 2B to E) unambiguously identified a differential expression pattern which correlates with the nuclear staining (grayscale image) and the stages of the cycle occurring within individual vacuoles. The parasites that are in G_1_ (based on the lack of daughter buds along with small tightly packed nuclei) had lower levels of TgOTUD3A-HA expression (G_1_, Fig. 2B) than did the ones that progressed through S phase, based on larger nuclear size and higher labeling intensity (S/M, Fig. 2B), and are at early cytokinesis as evidenced by the emergence of daughter buds (E, Fig. 2B). The parasites that are in the advanced stages of cytokinesis (I, Fig. 2C, and L, Fig. 2D) showed higher levels of TgOTUD3A-HA expression than did the ones in early cytokinesis or G_1_/S. Finally, a strong TgOTUD3A-HA signal was observed in the residual bodies (RBs), where all remaining maternal components are targeted after the daughters become separated from the mother (RB, Fig. 2D). We further measured the TgOTUD3A-HA signal intensity in the parasites from different stages of the cell cycle. The mean intensity of TgOTUD3A-HA signals appears to be significantly different (Fig. 2F and G) across the stages of the cell cycle and follows the transcript expression pattern as shown previously (17).

### TgOTUD3A is localized to the cytoplasm and partly colocalizes with developing daughter (TgIMC1) scaffolds.

The examination of the distribution of TgOTUD3A-HA in the tagged parasites reveals that TgOTUD3A is localized in the cytoplasm and distributed as distinct granular puncta (Fig. 2B to E). Of note, no significant nuclear staining was observed. The distribution of TgOTUD3A-HA in gravid parasites appears to be somewhat concentrated on early-, intermediate-, and late-stage daughter scaffolds (E/I/L, Fig. 2E) with additional labeling observed within the cytoplasm of daughters and the mother parasite. Notably, there appears to be no localization of TgOTUD3A-HA on the maternal scaffold. In the vacuoles where the residual bodies were found, TgOTUD3A-HA was also found to be localized to the residual bodies (RB, Fig. 2D and E).

### Recombinant TgOTUD3A has selective activity against polyubiquitin but not monoubiquitinated targets.

**FIG 3:**
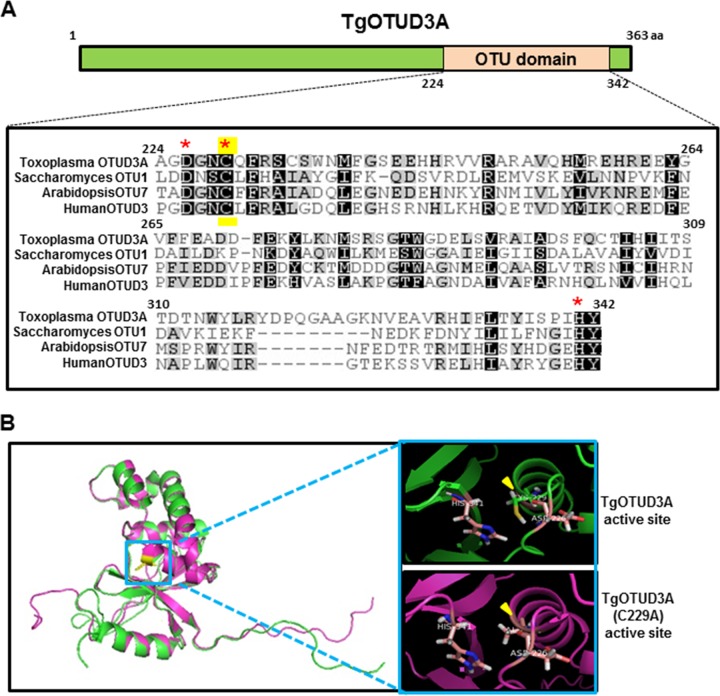
Sequence alignment and structure prediction of TgOTUD3A. (A) Schematic of TgOTUD3A structure, highlighting the position of the OTU domain. Amino acid alignment of TgOTUD3A (TGGT1_258780), human OTUD3 (sequence accession number NP_056022.1), *Saccharomyces* OTU1 (SGD accession number S000001850), and *Arabidopsis* OTU7 (sequence accession number AFS88955.1). Despite evolutionary distances, critical amino acid residues forming the catalytic triad (aspartate, cysteine, and histidine) are highly conserved across these species (red asterisks). The catalytic cysteine that was mutagenized to generate TgOTUD3A (C229A) is highlighted (yellow). (B) Predicted protein structures of wild-type TgOTUD3A and the TgOTUD3A (C229A) mutant were obtained in PDB (Protein Data Bank) file format using the RaptorX (http://raptorx.uchicago.edu/StructurePrediction) program and were viewed and analyzed using the protein modeling software PyMOL1.7.4.4 Edu (educational product) (Schrodinger, LLC). Structural alignment of the predicted wild-type (green) and mutant (purple) structures revealed no substantial difference in the overall structure or in the active site (insets). The yellow arrowhead highlights the spatial location of the mutagenized residue.

TgOTUD3A is predicted to be a member of the cysteine protease superfamily. Based on the sequence analysis, we have identified the amino acids (aspartate [D226], cysteine [C229], and histidine [H341]) in TgOTUD3A which are predicted to form the catalytic triad, a unique feature of all cysteine protease family proteins. We aligned the amino acid sequences of the OTU domain of TgOTUD3A with the closest human (OTUD3; GenBank accession number NP_056022.1), *Saccharomyces cerevisiae* (OTU1; *Saccharomyces* Genome Database [SGD] accession number S000001850), and *Arabidopsis thaliana* (OTU7; GenBank accession number AFS88955.1) orthologs and found TgOTUD3A to be 28.3%, 14.3%, and 30% identical, respectively, to these evolutionarily divergent species. Despite this poor overall homology, the critical amino acid residues of the catalytic triad were found to be highly conserved across these species (Fig. 3A). We expressed both the wild type and a catalytic mutant (C229A) form of recombinant TgOTUD3A full-length proteins in *Escherichia coli* for activity assays. Before examining the biochemical activity of the recombinant proteins, we performed a structure prediction analysis of both wild-type TgOTUD3A and its catalytic mutant (C229A) *in silico* using homology-driven protein modeling (PyMOL program). Our prediction analysis indicated that the catalytic cysteine mutation (C229A) should not have any effect on overall predicted protein folding (Fig. 3B).

**FIG 4:**
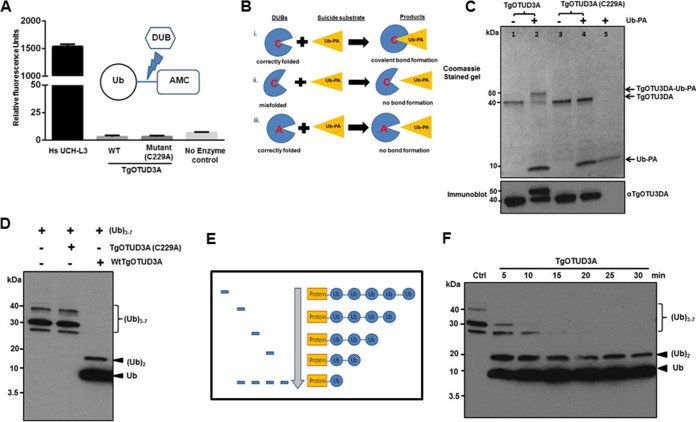
TgOTUD3A activity is directed specifically at polyubiquitin and is dependent on the catalytic cysteine (C229) in the active site. (A) Neither recombinant wild-type TgOTUD3A nor mutant TgOTUD3A (C229A) exhibited any activity against monoubiquitinated target in a fluorescence cleavage assay using an Ub-AMC substrate at 37°C for 1 h. Human HsUCH L3 was used as a positive control. (B) Schematic showing three possible scenarios to explain outcome of suicide substrate assay. (C) The ability of ubiquitin to bind to the wild-type or mutant protein was established using an electrophoretic mobility shift assay. Recombinant wild-type TgOTUD3A and the mutant TgOTUD3A (C229A) were incubated with suicide substrate (Ub-propargylamine at a 3 M excess). After a 30-min incubation, reaction mixtures were resolved by SDS-PAGE and stained with Coomassie blue (upper panel). Duplicate experimental samples for each reaction mixture were used to perform Western blotting using TgOTUD3A mouse polyclonal antiserum (lower panel). Both Coomassie blue staining and immunoblotting indicated that roughly 60 to 70% of wild-type TgOTUD3A forms a cross-linked adduct with the suicide substrate, resulting in a shift in mobility. This shift was not observed in the TgOTUD3A (C229A) mutant. (D) Deubiquitination assay with polyubiquitin chains (a mix of Ub trimers to heptamers) demonstrates the breakdown of the Ub polymer predominantly into the monomeric form with some dimers following incubation with TgOTUD3A for 15 min. The essentiality of the catalytic cysteine (C229A) is confirmed by the absence of any activity against poly-Ub. Monoubiquitin and polyubiquitin were detected using antiubiquitin antibody in immunoblot analysis. (E) Schematic showing the predicted pattern of time-dependent digestion of a polyubiquitin chain if the DUB is an exodeubiquitinase. The predicted pattern for an endodeubiquitinase would be a more random, nonsequential degradation. (F) Digestion pattern of polyubiquitin chains (trimer to heptamer) in time course incubation using a 5-min interval. The stair-like distribution, similar to that predicted in panel E, establishes that TgOTUD3A is an exodeubiquitinase.

We tested both recombinant wild-type and TgOTUD3A (C229A) mutant forms of the enzyme in an *in vitro* fluorometric deubiquitination activity assay (SenoLyte 520 DUB assay kit; AnaSpec). This assay works on the basis of the deconjugation of the ubiquitin from a quenched conjugated fluorophore, 7-amino-4-methylcoumarin (AMC), resulting in fluorescence emission from the freed AMC. Neither the wild type nor the cysteine mutant form of TgOTUD3A (C229A) showed any activity relative to either the no-enzyme control or the positive control (a human DUB; UHC L3) (Fig. 4A). This finding suggested three possibilities: (i) monoubiquitinated targets may not be the substrate of this enzyme, (ii) the enzyme needs to bind both ubiquitin and a specific region or scaffold of the substrate protein to cleave the conjugated ubiquitin, or (iii) alternatively the lack of activity could be attributed to improper folding of the recombinant enzyme.

To test if the purified recombinant enzyme was correctly folded, we performed a suicide probe assay where ubiquitin-propargylamine (Ub-PA; in which glycine-76 of ubiquitin was replaced by an alkyne moiety, propargylamine) was used as a probe (33). This alkyne probe forms a vinyl thioester bond with the catalytic cysteine of the enzyme if it is correctly folded and thus possesses a structurally competent substrate binding site (Fig. 4B). We used the Ub-PA probe in a 3 M excess for both the wild type and the catalytic mutant (C229A) of TgOTUD3A and found that the majority (approximately >70%) of the wild-type TgOTUD3A bound to the probe and made a covalent bond with the catalytic cysteine while the mutant (C229A) was unable to bind to the suicide substrate (Fig. 4C). This result clearly demonstrated that most of the recombinant wild-type enzyme was correctly folded and that its catalytic pocket was able to bind ubiquitin.

The absence of activity against a monoubiquitinated substrate led us to examine if recombinant TgOTUD3A can remove ubiquitin monomers from polyubiquitin. Recombinant wild-type TgOTUD3A was able to digest polyubiquitin chains to ubiquitin monomers (Fig. 4D), suggesting that it targets unanchored polyubiquitin or polyubiquitinated substrates. The deubiquitinase activity was crucially dependent on the integrity of the catalytic triad, as the mutant TgOTUD3A (C229A) enzyme was functionally inactive (Fig. 4D).

In order to determine if TgOTUD3A was an exo- or an endodeubiquitinase, we performed a time course experiment to establish the degradation pattern as a function of time. An exodeubiquitinase activity will generate a laddering pattern by removing ubiquitins sequentially one at a time from an end of a polyubiquitin chain (Fig. 4E), in contrast to the activity of an endodeubiquitinase, where the pattern of ubiquitin removal from the polyubiquitin chain would be more random**.** The observation of a time-dependent laddering pattern by sequential release of ubiquitin monomers indicates that TgOTUD3A is an exodeubiquitinase (Fig. 4F).

### TgOTUD3A exhibits a preference for specific lysine-linked polyubiquitin chains.

**FIG 5:**
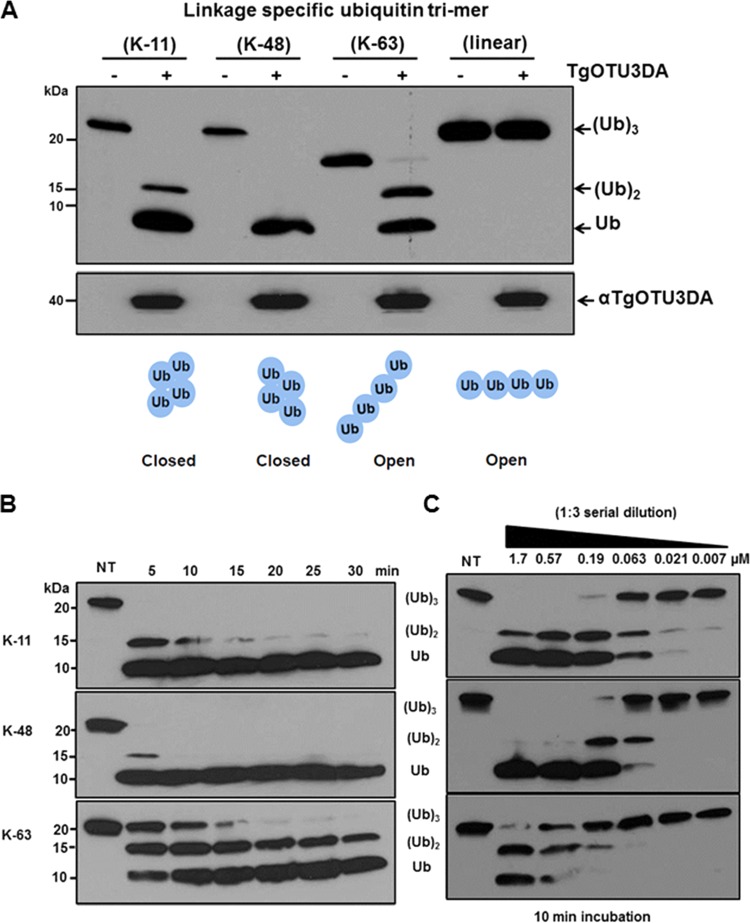
TgOTUD3A exhibits linkage specificity for different K-linked polyubiquitin chains. (A) A fixed amount of recombinant TgOTUD3A was incubated for 30 min with Ub trimers possessing different lysine-linked (K11, K48, and K63) chains or a linear Ub chain (head-to-tail polymer with the C-terminal Gly connected to the N-terminal Met). The reaction mixture was analyzed by immunoblotting. The first lane in each pair contains the linkage-specific substrate alone, while the second has recombinant TgOTUD3A added (see the lower panel for the immunoblot). Based on the relative levels of (Ub)_3_, (Ub)_2_, and Ub, TgOTUD3A exhibits a substrate preference for K48 relative to K11- and K63-linked poly-Ub. TgOTUD3A exhibits no activity toward end-to-end linear polyubiquitin chains. Below is a schematic representation of the established structural conformations of linkage-specific poly-Ub chains defining closed and open organization (37). (B) The specific linkage preference is confirmed using a kinetic analysis revealing a complete breakdown of K48-linked poly-Ub to monomer by a 10-min reaction. In the same time period, there were still undigested Ub dimers and Ub trimer present for the K11-linked and the K63-linked poly-Ub chains, respectively. NT, no treatment. (C) The effect of TgOTUD3A concentration on the breakdown of linkage-specific Ub trimers confirms the maximal activity against K48-linked poly-Ub relative to K11-linked substrate. Activity against K63-linked poly-Ub required considerably higher levels of TgOTUD3A. Together, these analyses confirm a substrate preference for K48- over K11- over K63-linked poly-Ub chains.

Ubiquitin has seven lysine residues (K6, K11, K27, K29, K33, K48, and K63) which can participate in forming isopeptide bonds with the terminal glycines (Gly-75 and -76) of another ubiquitin molecule (34). Involvement of a specific lysine in the formation of isopeptide bonds produces linkage specificity and structurally diverse branched-chain conformations (35, 36). In contrast, when the ubiquitins are conjugated in a head-to-tail fashion (between the initiator N-terminal methionine and the C-terminal glycine), a linear ubiquitin polymer chain is formed (35, 36). The specific lysine linkage of the polyubiquitin chains confers various conformations on the overall structure of the polymer. The K48- and K11-linked chains adopt a “closed” confirmation, while the K63-linked and linear chains present an “open” confirmation (36, 37) (Fig. 5A).

We tested the ubiquitin linkage-specific activity of recombinant TgOTUD3A against the unanchored synthetic ubiquitin trimer of the three most prevalent mammalian nonlinear chains (K48, K63, and K11) and a linear chain in an *in vitro* digestion reaction. In a 30-min reaction, recombinant TgOTUD3A digested ubiquitin trimers of different lysine-linked chains (K11, K48, and K63) into dimer and monomer with a different level of efficiency but was not able to digest the trimer of linear chains under the same reaction conditions (Fig. 5A). To further determine the precise linkage specificity of TgOTUD3A among these 3 nonlinear polyubiquitin chains, we performed a kinetic analysis by using a fixed amount of substrate (ubiquitin trimer) and a fixed amount of recombinant enzyme, stopping the reaction at 5-min intervals. We found that within 10 min, all K48-linked ubiquitin trimers were digested into ubiquitin monomers, whereas it took 30 min to have similar levels of accumulation of ubiquitin monomers from the breakdown of K11-linked ubiquitin trimers. Even in 30 min, a significant amount of ubiquitin dimers was present in the digestion of K63-linked chains (Fig. 5B). To confirm this result, we tested the activity preference in another assay in which we used a fixed concentration of the ubiquitin trimer (1 µg) and different dilutions of the enzyme for determining the efficiency of the enzymes in a 10-min reaction. At a 1:3 dilution (0.57 µM), TgOTUD3A digested all the K48-linked ubiquitin trimers to monomers within 10 min. In the same dilution range, the reactions with the K11-linked chains and K63-linked chains had significant accumulation of undigested dimers and trimers, respectively (Fig. 5C). The efficiency of digestion from dimer to monomer is also higher for the K48- than for the K11-linked chains, although at a 1:9 dilution (0.19 µM), the reaction kinetics appear to be similar for K48-linked and K11-linked chains. These results are in agreement with the findings of a previous activity preference assay (Fig. 5A and B). Altogether, we have determined that the preference for linkage-specific activity of TgOTUD3A is K48 > K11 > K63. This biochemical activity profile of TgOTUD3A is different from that of its structural human ortholog, *Homo sapiens* OTUD3 (HsOTUD3) (Table 1) (26).

### *T. gondii* proteins are modified by linkage-specific ubiquitination.

**FIG 6:**
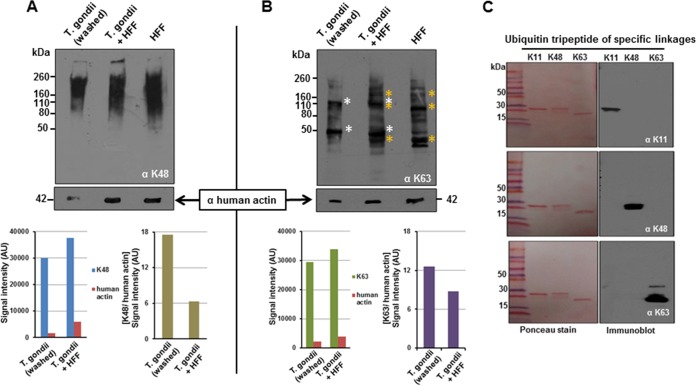
Evidence for diverse lysine-linked ubiquitin-modified proteins in *T. gondii*. Washed and unwashed parasites (5 × 10^6^) and an equivalent volume of uninfected HFF lysate were subjected to immunoblot analysis using anti-K48 and -K63 linkage-specific antibodies. Following exposure, the blots were stripped and reprobed with an anti-human actin antibody to assess the level of host contamination in the parasite-containing samples. Immunoreactivity signals for linkage-specific ubiquitin antibodies (K48 and K63) and anti-human actin antibody were measured by using the ImageJ (NIH) gel lane analysis tool in arbitrary units (AU) for the first two lanes (washed and unwashed parasites) to provide a semiquantitative measure of relative host contamination in the parasite-containing samples. (A) K48 blot. Due to the higher diversity of the K48 linkage in both the parasite (*T. gondii* washed and *T. gondii* + HFF) and the host cell (HFF), the pattern appears as a high-molecular-weight smear. The distribution of the smear in the washed parasites, however, is different relative to the samples containing more host contamination. The level of host contamination is estimated using an anti-actin antibody. In order to determine the level of host contamination, the total signal per lane and the K48 signal relative to actin were established for the parasite-containing samples. These data confirm that the bulk of the K48 signal in the washed sample is from K48-modified parasite proteins. (B) K63 blot. The diversity of K63 linkage-modified parasite proteins is significantly lower than that of K48 (distinct bands indicated by white asterisks in *T. gondii* washed and *T. gondii* + HFF). HFF-derived bands are indicated by yellow asterisks. The level of host contamination and level of parasite-specific signal were determined as described above. (C) As an antibody specificity control, synthetic ubiquitin trimers of different linkages were resolved by SDS-PAGE and separately probed with K11, K48, and K63 antiubiquitin linkage-specific antibodies. All three antibodies detected the specific linkages with no cross-reactivity.

The diversity of polyubiquitin linkages present in *T. gondii* is not known. The TgOTUD3A activity profile suggests the existence of specific K-linked polyubiquitin in *T. gondii*. In order to establish the presence and diversity of polyubiquitin modifications in *T. gondii*, we used an immunoblotting approach with ubiquitin linkage-specific antibodies. To minimize contamination from ubiquitinated host proteins, we extensively washed the parasites following syringe passage. The total number of parasites following the washing was determined, and a volume corresponding to 5 × 10^6^ parasites per lane was used. We also used 5 × 10^6^ parasite equivalents from our unwashed sample and the volume equivalent from the uninfected HFF cells. These samples allow us to roughly distinguish between host- and parasite-derived signals, as the linkage-specific antibodies do not inform us of the origins of the modified proteins. As was previously reported for human cells (38, 39), the signal for K48-modified proteins appeared as a broad high-molecular-weight smear in the HFF-only sample. This general pattern is also observed in an unwashed infected-cell sample (Fig. 6A). In contrast, for the washed parasite sample, the K48-linked smear pattern was more concentrated at a higher molecular weight range (Fig. 6A). In order to confirm that the washing effectively removed host material, we stained the immunoblot with anti-human actin. The intensities of the actin band and the K48 signal in their respective lanes were measured and plotted as total signals or as the ratio of K48 to actin within each infected sample (Fig. 6A). This ratio confirms that most of the K48 signal in the washed sample is of parasite origin.

The diversity of K63-linked proteins in the parasite extract, which appear as discrete bands, is considerably lower than that of K48-linked proteins (Fig. 6B). The presence of distinct bands in the infected samples (washed and unwashed) that are not evident in the uninfected HFFs points to specific parasite proteins being K63 linked (Fig. 6B). This is supported by the quantification of the overall K63 signal and the intensity relative to host actin (Fig. 6B).

We did not detect any K11-specific antibody staining either in the parasite or in the host cells by immunoblotting, even when 1 × 10^8^ parasite equivalents were probed in the immunoblot assay (data not shown). This is likely due to the possibility that K11-specific ubiquitin modification appears in the parasite in a certain narrow window of the cell cycle, as is the case for human cells, where the K11 linkage is restricted to mitotic cells (39). The frequency of mitotic cells in contact-inhibited HFFs is typically under 5% of the population (40). Of note, the anti-K11 antibody specifically reacts with synthetic K11-linked ubiquitin chains (Fig. 6C). The specificity of the K48- and K63-specific antibodies was similarly confirmed (Fig. 6C).

**FIG 7:**
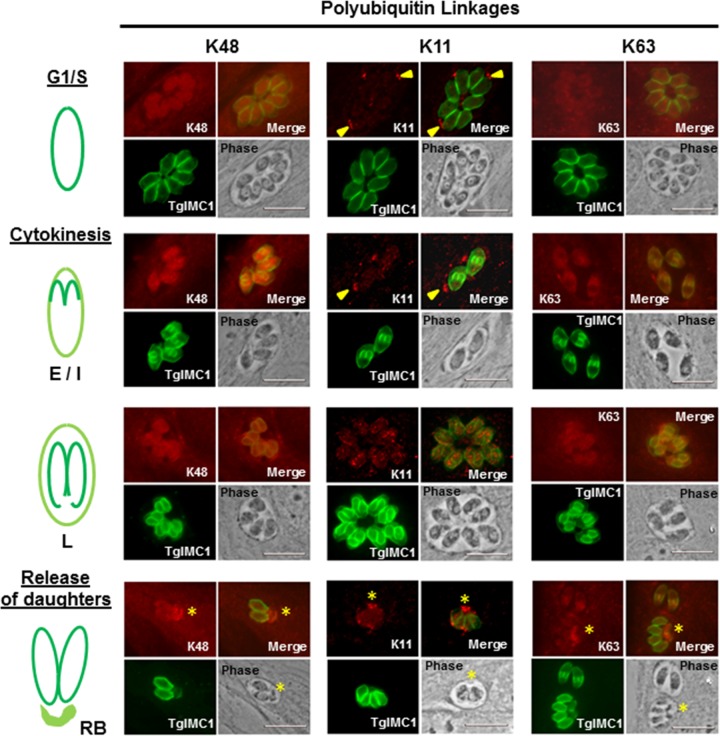
Levels and localization of specific ubiquitin linkage modifications in *T. gondii* at different stages of endodyogeny. An indirect immunofluorescence assay using K48, K11, or K63 linkage-specific antibodies relative to endodyogeny using anti-TgIMC1 was performed. (Left) Staining with anti-K48 antibody in the G_1_ parasites is brighter than that of host cells and is uniform over both cytoplasm and nucleus. The intensity of the K48 signal appears brighter in the parasites in the early stage during cytokinesis (E/I) but appears to decrease as the daughter scaffolds mature (L and release of daughters) and finally becomes concentrated in the residual body (yellow asterisk). (Middle) No significant K11 signal (red) within G_1_ parasites. Patches of K11 labeling are sometimes found in the vacuolar space outside the parasite (yellow arrowheads). No significant host cell staining was observed. Parasites with immature daughter buds (E/I) exhibit a signal marginally above that of G_1_ parasites, which lack daughter buds. K11 signal increased significantly in the parasite with mature daughter buds (L) where the signal appears to be punctate and with no specific localization. The K11 signal becomes more diffuse as the daughters are separating from the mother, ending up concentrated in the residual body (yellow asterisk). (Right) The anti-K63 antibody signal appears to be diffuse and barely detectable above the host cell background in G_1_/S parasites. Both the levels and distribution of the anti-K63 signal change with developing daughter buds as it becomes more concentrated toward the anterior of the parasite where the daughter buds are developing (E/I). The K63-linked modification exhibits a more distributed and diffuse pattern relative to the late-stage daughter scaffolds (L). The K63 modification becomes less intense in the recently emerged daughters (release of daughters) with the signal redistributing to the residual body (yellow asterisk). Bars, 10 µm.

To further confirm these immunoblotting results and to localize the linkage-specific modification in the parasite *in vivo*, we performed indirect immunofluorescence assays (IFAs). In these IFAs, we used TgIMC1 as a guide for different stages of endodyogeny. All three ubiquitin linkage-specific antibodies (K11, K48, and K63) showed a range of staining intensities and distributions in the parasite at various stages of the cell cycle (Fig. 7). The K48-specific signal was found in both the cytoplasm and the nucleus. The intensity of the signal increased as the parasite cell cycle progressed through mitosis and early cytokinesis (E/I, Fig. 7, left panel). As the daughters matured within the mother (defined by the size of the daughter scaffolds), K48-linked protein intensity decreased (L, Fig. 7, left panel) and was accompanied by an accumulation of K48-associated signal within the residual body when the daughter parasites separated from the mother (RB, Fig. 7, left panel).

Levels of K63-linked modifications were very low during G_1_/S and gradually increased as the daughter buds emerged (G1/S/E/I, Fig. 7, right panel). We further observed a concentration of K63-linked ubiquitin-modified targets in the vicinity of developing daughter buds (E/I, Fig. 7, right panel). As the daughters progressed through the maturation process, the K63 polyubiquitin linkage signal became more diffuse throughout the cytoplasm, finally concentrating in the residual body (RB, Fig. 7, right panel).

The K11-linked polyubiquitin modification was barely detectable in the parasites at G_1_/S or at early stages of cytokinesis (G_1_/S/E/I, Fig. 7, middle panel), which was consistent with the absence of any signal shown by immunoblotting (data not shown). The punctate K11 signal was detected throughout the cytoplasm at later stages of endodyogeny (L, Fig. 7, middle panel). The concentration of the K11-specific signal in the residual body further suggests that the residual body is the major “recycling bin” for the proteins, given its connection to the ERAD pathway (41) (RB, Fig. 7, middle panel). In some instances, the K11 signal was present in the parasitophorous vacuole (G_1_/S, Fig. 7, middle panel). This pattern, which likely represents the debris from broken-down residual bodies, was not detected with antibodies specific for K48 and K63 linkages (Fig. 7). Thus, despite the lack of a signal for K11-modified proteins in the immunoblot analysis, its detection within individual parasites at the later stages of the cell cycle suggests a highly specific function(s).

**FIG 8:**
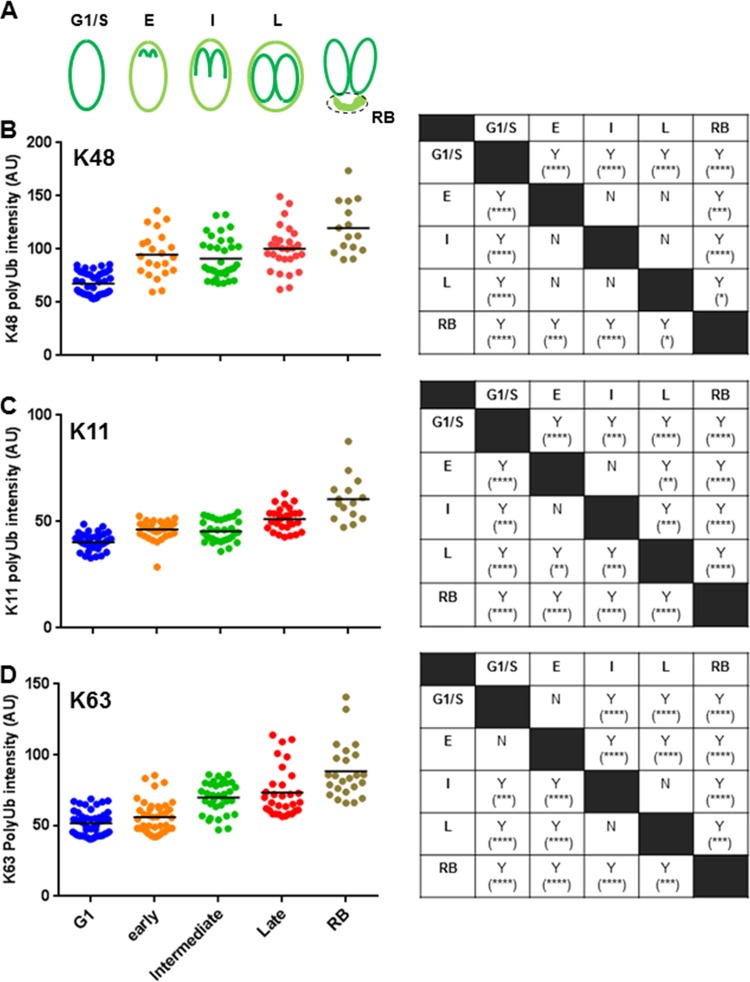
Quantification of linkage-specific (K48, K11, and K63) polyubiquitin signals across the stages of the *T. gondii* cell cycle and in the residual body (RB). (A) The cartoon shows how the pattern of TgIMC1 signal was used as a guide to categorize different stages of the cell cycle in which specific polyubiquitin signal intensity of the whole parasite was measured. (B) The images of K48 polyubiquitin antibody-stained parasites were captured at random at a fixed exposure time. The intensity per unit area of the parasite was measured by ImageJ software and plotted to show the distribution and range of signal intensity at different cell cycle stages. For the residual body category, only the signal intensity of the residual body was measured. One-way ANOVA shows a significant difference between the cell cycle groups (*F*_4,136_ = 27.47 [*P* < 0.0001]). (C and D) Similarly, images were captured for K11 (C) and K63 (D) polyubiquitin signals and measured by ImageJ software. One-way ANOVAs show significant differences of signal intensity between different cell cycle groups of parasites both for K11-linked (*F*_4,158_ = 48.23 [*P* < 0.0001]) and for K63-linked (*F*_4,178_ = 41.72 [*P* < 0.0001]) polyubiquitin. The analyses between cell cycle stages were done using Tukey’s pairwise multiple-comparison test (α = 0.05). The mean fluorescence intensities which differed significantly are indicated by Y (for yes), and the level of significance is indicated by the number of asterisks. The letter N (for no) is used to denote that no significant difference existed between groups.

We further quantified the fluorescence intensity of the specific ubiquitin linkages to establish whether cell cycle-associated patterns would emerge by using TgIMC1 as a marker to determine the approximate stages of the *T. gondii* cell cycle (Fig. 8A). The quantification of all three linkage-specific polyubiquitin signals and analyses show that the levels of K48-, K11-, and K63-linked polyubiquitin modifications vary significantly across the cell cycle (Fig. 8B to D). Based on the overall dynamics, semiquantitative measurements of linkage-specific ubiquitin modification, and the antibody sensitivities (see Materials and Methods), we came to the conclusion that the most abundant nonlinear ubiquitin modification in the parasite is the K48 linkage, followed by the K63 and K11 linkages, respectively, among the three polyubiquitin linkages that we tested.

### TgOTUD3A is able to digest K48-linked but not K63 ubiquitin-modified proteins of *T. gondii.*

**FIG 9:**
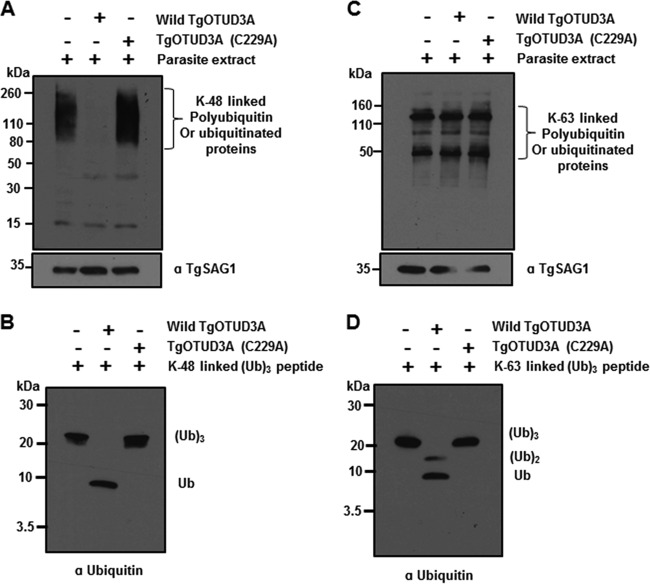
TgOTUD3A selectively deubiquitinates K48-linked *Toxoplasma* proteins. Washed parasites were mechanically lysed and incubated in the DUB reaction buffer without (control) or with either recombinant wild-type TgOTUD3A or mutant TgOTUD3A (C229A) enzyme in a 30-min reaction, resolved using SDS-PAGE, transferred, and probed with linkage-specific antibodies. (A) K48 linkage-specific antibody shows the removal of K48-linked ubiquitin *T. gondii* proteins in the presence of the wild type but not its cysteine (C229A) mutant. Notably, all K48-linked ubiquitin modifications are removed from protein targets by the wild-type enzyme. (B) The same enzyme mix used in panel A was tested using the synthetic K48 polyubiquitin chains, confirming activity in the wild-type enzyme and no activity in the C229A mutant. (C) The same parasite extract and enzyme used in panel A were resolved and probed with a K63 linkage-specific antibody. Neither wild-type nor mutant enzyme was able to digest *T. gondii* K63-linked ubiquitinated proteins, confirming that K63-linked polyubiquitin is a poor substrate for TgOTUD3A. (D) Enzyme and buffer controls using the synthetic K63-linked substrate confirm partial activity. Both digestion blots were stripped and reprobed with TgSAG1 antibody for a loading control (A and C).

Using synthetic unanchored ubiquitin tripeptide of different linkages, we have shown above that TgOTUD3A has a preference for specific K-linked ubiquitin modification (Fig. 5), which serves as indirect evidence that the substrate preference of TgOTUD3A is for K48-linked or K11-linked ubiquitin chains. Our ability to label K11-, K48-, and K63-linked ubiquitinated *T. gondii* proteins with the linkage-specific antibodies led us to examine whether recombinant wild-type TgOTUD3A can digest *T. gondii* proteins which are modified by any of these 3 nonlinear polyubiquitin chains. We incubated recombinant wild-type TgOTUD3A or the mutant TgOTUD3A (C229A) enzyme with a mechanically lysed parasite extract to establish whether it could deubiquitinate the ubiquitin-modified *Toxoplasma* proteins in the washed parasite extract (less host contamination). In a 30-min incubation reaction, wild-type TgOTUD3A digested almost all the K48-linked ubiquitin modifications on diverse parasite proteins (observed as a smear in Fig. 9A) but failed to digest any K63-linked modification (Fig. 9C). It is important to note here that the washed parasite extract is likely to have some host K48-linked proteins. Complete digestion of all modified proteins indicates that the recombinant enzyme removed the K48-linked ubiquitin irrespective of the target proteins. In addition, the C229A catalytically inactive enzyme failed to deubiquitinate any modified proteins regardless of the linkage specificity. The observed activity against the synthetic substrate confirmed that the assay condition and the enzyme activity were optimum (Fig. 9B and D). We could not perform the same assay for K11-linked parasite proteins because of their low abundance, which makes them difficult to detect on the immunoblot (data not shown).

Together, the demonstration of diverse polyubiquitin modification in *Toxoplasma gondii* and the selective degradation of polyubiquitin by a deubiquitinase, TgOTUD3A, can provide a basis for fine-tuned functional regulation during the cell cycle and normal homeostasis.

## DISCUSSION

The replication of *T. gondii* by endodyogeny is unique and complex in the aspects that the generation of daughter parasites occurs within the mother while the maternal components are being turned over (5). This necessitates spatiotemporal regulation by the selective destruction of maternal structures while protecting the essentially identical structures in the developing daughters. We hypothesized that the ubiquitin-mediated selective degradation and its fine-tuned regulation by deubiquitinases play critical roles in this highly regulated process. The important role of proteasome-mediated degradation in *T. gondii* has been demonstrated, as the inhibition of the proteasome severely disrupts multiple stages of the replication (42). While a few studies describe apicomplexan ubiquitin ligases (15, 20), only one study to our knowledge has looked into a deubiquitinase in *T. gondii* (19). None of these studies investigated the role of ubiquitin-mediated regulation of apicomplexan replication. A recent release of the *T. gondii* ubiquitome (16) reveals that a number of ubiquitinated proteins are also regulated at the transcriptional level in a cell cycle-dependent manner.

In this study, we have characterized an OTU family deubiquitinase which is transcriptionally regulated during *T. gondii* cell cycle progression. The role of OTUs in *Drosophila* germ cell proliferation (23, 43) and various human cancers (24, 25, 44) hints at their role in cell cycle regulation. The role of OTU DUBs (OTUs) in apicomplexans has remained unexplored. The *T. gondii* genome encodes the largest number of OTU domain-containing proteins among the sequenced apicomplexans (15). The number of predicted OTUs encoded in the *T. gondii* genome is comparable to numbers in the much larger human and *Arabidopsis* genomes (26, 45). In contrast, *Saccharomyces cerevisiae* possesses only two OTUs (46). Consistent with the human and *Arabidopsis* OTUs, *Toxoplasma* OTUs are distributed in different phylogenetic clusters. The unique OTU domain structure of some members and the presence of homologous OTU domain-containing members in different phylogenetic clusters suggest a possible adaption of diverse function.

TgOTUD3A is distinct from other OTU family members as it exhibits a very dynamic cell cycle-dependent transcript expression profile and its *Plasmodium* ortholog is expressed only at the “schizont stage” in red blood cells (22). Expression of epitope-tagged protein mirrored the mRNA expression profile (17), indicating that TgOTUD3A protein expression is controlled primarily at the level of transcription. The selective concentration of HA-tagged protein on the daughter scaffolds during endodyogeny suggests a potential protective mechanism to prevent the degradation of the newly formed daughter scaffolds during a period of high overall turnover of maternal inner membrane complexes (IMCs) (47). This is quite interesting in the light of recent findings by Silmon de Monerri et al. (16) that 18% of the *T. gondii* ubiquitome consists of proteins associated with the inner membrane complex. As the cytoskeletal proteins are constitutively remodeled during the progression of the cell cycle, a deubiquitinase such as TgOTUD3A might be important to regulate spatiotemporal turnover of maternal IMCs while protecting the daughter IMCs inside the mother.

TgOTUD3A is predicted to be a member of the cysteine protease family of DUBs. Our data have clearly shown that TgOTUD3A is a true cysteine protease and functions as an exodeubiquitinase which can remove ubiquitin from nonlinear polyubiquitin chains. The loss of DUB activity due to a point mutation of the conserved catalytic cysteine (C229A) is consistent with the activities of both human and *Arabidopsis* OTUs (26, 45), indicating a high level of evolutionary conservation. The activity preference of TgOTUD3A toward polyubiquitin linkages (K48 > K11 > K63) can be explained structurally by the “closed” conformation of both K48 and K11 chains (34, 36, 37, 48, 49) as opposed to the “open” conformation of K63-linked and linear chains (36, 50). The conformation of these different nonlinear polyubiquitin chains is dependent on which of the 7 encoded lysine residues participates in the formation of the “isopeptide bond” between two linked ubiquitin molecules (34, 36). Polyubiquitin chains in solution exhibit structural flexibility (36), permitting a DUB to cleave a nonspecific linkage at a higher concentration (26). This explains the secondary specific activities of the OTUs (26, 45), as is the case for the lower activity of TgOTUD3A against the K63-linked chains. The ability of the recombinant TgOTUD3A to remove ubiquitin from K48-linked but not K63-linked ubiquitin-modified *T. gondii* proteins provides a direct confirmation that this deubiquitinase is very specific to digestion of K48-linked ubiquitin modification in the context of ubiquitinated targets in *T. gondii*. However, the complete digestion of K48 linkages from *T. gondii* proteins suggests that TgOTUD3A recognizes primarily the K48-linked ubiquitin chain as opposed to the ubiquitinated protein targets, as is the case for some of the highly selective DUBs (51).

The connections between the linkage specificity and functional activity of OTUs do not always appear to be conserved. For example, TgOTUD3A is structurally the closest ortholog of human OTUD3, which has preferential activity for K6- and K11-linked polyubiquitin chains (26). However, based on our biochemical characterization, TgOTUD3A is functionally closest to the human OTU protein VCPIP, which has specificity for K11- and K48-linked chains (26). Interestingly, our bioinformatic analysis did not identify any VCPIP-like OTU domain-containing OTU in *T. gondii*.

In mammalian cells, out of seven possible ubiquitin chain linkages, 3 chains (K48, K63, and K11) are the most prevalent (38). The polyubiquitin linkages on proteins serve as a code to determine the fate of protein substrates, affecting their turnover, localization, and functions (34). Among the 3 nonlinear chains, two polyubiquitin chains (K48 and K63) (49, 50) are found to be involved with 2 distinct functions: the K48-linked polyubiquitin chains are the canonical signature of proteasome-mediated degradation (52) while substrates conjugated with the K63-linked polyubiquitin chain are normally associated with nondegradative pathways such as vesicular trafficking, endocytosis, and cell signaling (52) and are also shown to be involved in autophagy (53), a lysosome-mediated bulk degradation and recycling process (9). In recent years, the K11-linked chains have emerged as important regulators of cell division in addition to other functions such as ERAD (endoplasmic reticulum-associated protein degradation) and signaling pathways (38, 41, 54). In contrast to mammalian species, K48-linked chains are involved in cell cycle regulation in yeast (55). This switch from K11 to K48 linkages for cell cycle regulation has been attributed to “closed” mitosis (nuclear envelope does not dissolve during mitosis) of yeast as opposed to the “open” mitosis (nuclear envelope disintegrates before chromosomal segregation) (41). *Toxoplasma* and other apicomplexans undergo “closed” mitosis (5), suggesting a potential functional similarity to yeast. Therefore, the cell cycle-associated expression and the K48- and K11-specific deubiquitinase activity of TgOTUD3A suggest its potential involvement in cell cycle regulation.

Our immunofluorescence analyses clearly indicate that *T. gondii* proteins are modified by different lysine-linked ubiquitin modifications. The levels and spatiotemporal accumulation of these different ubiquitin modifications are very dynamic during different stages of endodyogeny, suggesting their important role in parasite biology and replication. The high diversity and ubiquitous distribution of K48 ubiquitin modification in both the nucleus and the cytoplasm as opposed to the concentrated nuclear signal of total ubiquitin found by Silmon de Monerri and colleagues (16) suggest that this linkage may serve as a general degradation signal. In addition, as noted above, the nuclear localization of the K48 ubiquitin modification might suggest potential cell cycle-regulating activities as observed for yeast (55). The accumulation of K63 linkage in the vicinity of newly emerging daughter buds is suggestive of a potential role of K63-mediated vesicular transport (52) of the component proteins’ organellar biogenesis which needs to be further investigated. The detection of punctate K11 signal at later stages of endodyogeny suggests a possibility that K11 modifications may not be specific for mitotic activity in *T. gondii* but rather could be involved in other processes such as ERAD pathways as found in yeast (41). The absence of nucleus-specific K11 signal may argue against any specific role in mitosis. Interestingly, maternal remnants in the residual body label strongly with all three ubiquitin linkage-specific antibodies (K11, K48, and K63), suggesting a potential function associated with the turnover of excess maternal components. Moreover, the differential distribution and the dynamics of linkage-specific ubiquitination correlate with the profile of a linkage-specific deubiquitinase, TgOTUD3A, across the cell cycle. This is very intriguing in terms of tight spatiotemporal regulation and needs to be further investigated.

The work presented here strongly suggests that TgOTUD3A expression and localization and activity profile are associated with the cell cycle-related functions. Interestingly, the TgOTUD3A knockout (TgOTUD3A-KO) is viable, suggesting compensatory changes (data not shown). Though viable, TgOTUD3A-KO parasites do exhibit a complex phenotype associated with the fidelity of parasite replication which we are currently in the process of elucidating (data not shown). This suggests that the ubiquitination may play an important role in the fine control of endodyogeny in addition to the effects associated with bulk protein turnover.

Extensive research on ubiquitin-mediated pathways over the past 2 decades has expanded our knowledge about the role of ubiquitin-mediated posttranslational modifications not only in protein degradation but also in other diverse cellular processes (56). Our understanding of the “ubiquitin code” in Apicomplexa is still in its infancy. This study opens a new dimension to our understanding by demonstrating not only the presence of diverse linkages necessitating a large number of Ub-ligases and DUBs in *T. gondii* but also tying their dynamics to the progression of the cell cycle. Finally, with our characterization of TgOTUD3A we establish an experimental template for the functional biochemical characterization of *T. gondii* DUBs as a prelude to studies on how they impact both generalized and highly specialized parasitic processes.

## MATERIALS AND METHODS

### Parasite culture and maintenance.

Type I RH *ΔHXGPRTΔku80* and *ΔHXGPRTΔku80* wild-type TgOTUD3A-HA *Toxoplasma gondii* lines were cultured in primary human foreskin fibroblasts (HFFs; ATCC) as described elsewhere (9). HA-tagged parasites were selected in medium supplemented with 1 µM pyrimethamine (Sigma).

### Bioinformatic analysis of TgOTUs.

Putative TgOTUs were identified using text and OTU domain motif (Pfam accession number PF02338) searches (27) in the *Toxoplasma* genome database (http://www.toxodb.org). The putative hits were further verified for OTU domain and other structural features by BLAST searches in the EMBL Pfam database (http://pfam.xfam.org/), SMART (http://smart.embl-heidelberg.de/), and PROSITE (http://prosite.expasy.org/) analysis program. Conservation of active site residues was demonstrated by MUSCLE alignment, and the phylogenetic tree was drawn with the UPGMA (unweighted pair group method using average linkages) tree building method using Geneious version 6.1.8 (57). Predicted structures of wild-type and catalytically inactive TgOTUD3A (C229A) were determined using the primary amino acid sequences on a template-based protein structure modeling program, RaptorX (http://raptorx.uchicago.edu/StructurePrediction) (58). The structure alignment analysis and labeling of active site residues were done by using PyMOL1.7.4.4 Edu (Schrodinger, LLC).

### Epitope tagging of TgOTUD3A.

Genomic DNA 2.5 kb upstream of the second-to-last codon of the TgOTUD3A gene (TGGT1_258780) was amplified using forward and reverse primers anchored with ligation-independent cloning (LIC) adapter sequences (forward, 5′-TACTTCCAATCCAATTTAGCGCCTCGTCTTCCTTCG-3′; reverse, 5′-TCCTCCACTTCCAATTTTAGCCCCTCGTTGTGCGCCCGT-3′) and cloned into LIC cloning sites of the pHA_3x_LIC tagging plasmid (a kind gift from Peter Bradley, UCLA). Approximately 20 µg of NsiI-digested linearized plasmid was transfected in the RHΔku80 strain and selected with 1 µM pyrimethamine 24 h posttransfection. Parasites were maintained in medium containing 1 µM pyrimethamine. Drug-resistant parasites were screened for HA expression in IFAs and Western blot analyses, and HA-tagged parasites were clonally selected in a 96-well plate by limiting dilution.

### Immunofluorescence assay.

An HFF monolayer was grown in a confluent monolayer on coverslips in a 24-well plate and infected (1 × 10^4^ per well) with either RHΔku80 or TgOTUD3A-HA parasites. At 16 to 24 h postinfection, the infected cell monolayer was fixed in methanol as previously described (9). The following primary antibodies were used at the indicated dilutions: rabbit anti-HA (1:100; Cell Signaling; catalog no. 3724), mouse monoclonal antibody (MAb) 45:36 anti-TgIMC1 antibody (1:1,000) (30, 59), rabbit antiubiquitin (1:2,000; Millipore), rabbit antiubiquitin K11 linkage (1:100; 2A3/2E6; Millipore; catalog no. MABS107-I), rabbit antiubiquitin K48 linkage (1:100; Apu2; Millipore; catalog no. 05-1307), rabbit antiubiquitin K63 linkage (1:100; Abcam; catalog no. ab179434). IFA slides were visualized using a Zeiss AxioVision microscope with a 100× 1.4-numerical-aperture (NA) oil immersion objective and acquired using a high-resolution grayscale Zeiss AxioCam MRM digital camera. Images for anti-HA or anti-linkage-specific ubiquitin antibody signals were recorded at a fixed exposure time (for each antibody) to permit comparison of intensity levels at various stages of the parasite cell cycle guided by the anti-TgIMC1 staining profile. To generate representative images for each cell cycle stage, grayscale images were pseudocolored to reflect the emission of the specific fluorophore, Brightness and contrast were adjusted, and the merged images were generated using Adobe Photoshop CS6. All manipulations to acquired images (brightness/contrast) were applied uniformly to the presented images.

### Quantification of IFA signal intensity and statistical analysis.

For quantification of TgOTUD3A-HA signal or linkage-specific polyubiquitin signals, images were acquired at random and at a specific exposure time for each antibody using a Zeiss AxioVision microscope with a 100× 1.4-NA oil immersion objective and acquired using a high-resolution grayscale Zeiss AxioCam MRM digital camera. The intensity of the signal from the raw unmodified images was measured per unit area of the parasite or residual body by NIH ImageJ software. All the graphs for signal intensity quantification data and one-way analyses of variance (ANOVAs) were done using GraphPad Prism version 6.0 (La Jolla, CA). The *P* values were calculated, and the difference between any two group means was analyzed as described previously (28).

### Immunoblotting for detection of linkage-specific ubiquitin modifications.

Equivalent amounts of parasites (2 × 10^7^) either washed (minimum host contamination) or mixed with the host cells or the same amount of uninfected host cells was lysed with a French press. A one-fourth amount was loaded per lane (5 × 10^6^) and immunoblotted with a 1:100 dilution of each linkage-specific antibody. The sensitivity and the specificity of the antibodies were determined by loading the same amounts of synthetic peptides of different linkages and treating parasites with an individual linkage-specific antibody of the same dilution. The linkage-specific antibodies were very specific and showed no cross-reactivity for other linkages. Their sensitivities appear to be similar based on the comparative analysis of Ponceau staining and the immunoreactivity pattern.

### Cloning, site-directed mutagenesis, expression, and purification of TgOTUD3A.

Full-length coding sequence (single exon, open reading frame [ORF] of 1,092 bp) of TgOTUD3A (TGGT1_258780) was amplified by PCR from RH strain cDNA using forward and reverse primers (forward, 5′-CACCATGCCGCTCTGTCGGAACTC-3′; reverse, 5′-TCACCCTCGTTGTGCGCC-3′) and cloned into Champion pET100 vector (Life Technologies). The codon for the catalytic cysteine residue was mapped and mutagenized to alanine (C229A) by inverse PCR using a primer pair (forward, 5′-GGGCGACGGGAACGCCCAGTTCCGGTCC-3′; reverse, 5′-GGACCGGAACTGGGCGTTCCCGTCGCCC-3′) and the QuikChange II site-directed mutagenesis kit (Agilent Technologies). Both wild-type TgOTUD3A and mutant TgOTUD3A (C229A) protein expression plasmids were transformed into BL-21 Star (DE3) *E. coli*. For protein expression, transformed bacteria were grown to an optical density at 600 nm (OD_600_) of 0.8 at 37°C and induced with 500 µM IPTG (isopropyl-β-d-thiogalactopyranoside) for 16 to 20 h at 20°C in LB-ampicillin medium. Bacteria expressing wild-type and mutant 6×His-TgOTUD3A protein were lysed using a French press (10,000 lb/in^2^) and incubated with 250 U of Benzonase at room temperature (RT) for 15 min. Following sonication and centrifugation (13,000 rpm for 5 min), the supernatant was incubated with MagZ beads (Promega MagZ protein purification system) and eluted with 1 M imidazole. The purified protein was stored at −20°C in 30% glycerol.

### Generation of anti-TgOTD3A mouse polyclonal antiserum.

Four BALB/c mice were immunized with purified recombinant TgOTUD3A protein (first dose, 45 µg/mouse) with Freund’s complete adjuvant (Sigma; catalog no. F-5881), followed by 3 boosts (dose, 25 µg/mouse) in incomplete Freund’s adjuvant (Sigma; catalog no. F5506) at days 14, 21, and 49. On day 56, mice were sacrificed by CO_2_ asphyxiation followed by cervical dislocation in accordance with the protocol approved by the University of Kentucky IACUC. The raw serum was collected and tested by Western blotting and IFAs. The resultant polyclonal antiserum worked only in immunoblotting and not in IFA applications.

### Deubiquitination assays. (i) Fluorometric deubiquitination assay.

The fluorometric deubiquitination assay was performed using ubiquitin-AMC (7-amino-4-methylcoumarin) fluorophore (SensoLyte 520 deubiquitination assay kit; AnaSpec). Ubiquitin-AMC substrate was incubated with or without recombinant wild-type and mutant TgOTUD3A (C229A) enzymes. DUB activity of the recombinant proteins was measured according to the kit protocol.

### (ii) Linkage-specific deubiquitination assay.

The linkage-specific deubiquitination assay was performed as follows. Recombinant wild-type and TgOTUD3A (C229A) enzymes were activated and incubated with parasite extract or synthetic polyubiquitin substrate mix containing 1 µg of polyubiquitin chain (trimer or 3- to 7-polymer or trimer of specific linkages from Boston Biochem) diluted in 2× DUB buffer (100 mM NaCl, 100 mM Tris, pH 7.4, and 10 mM dithiothreitol [DTT]) and incubated at 37°C for indicated time points as described by Mevissen and colleagues (26). The reactions were stopped by addition of 2× Laemmli SDS-PAGE sample buffer, and reaction mixtures were run on a 4 to 20% Tris-glycine gradient gel (Bio-Rad). The proteins were transferred to a nitrocellulose membrane immunoblotted with anti-rabbit monoclonal ubiquitin primary antibody (dilution, 1:2,000; EMD Millipore; catalog no. 04-454) and goat anti-rabbit horseradish peroxidase (HRP) secondary antibody (dilution, 1:2,000; Thermo Scientific; catalog no. 31460) and developed on X-ray film (Kodak) with Super Signal West Pico chemiluminescence substrate (Thermo Scientific).

### Suicide substrate assay.

The suicide substrate assay was done as described earlier (26, 33) with some modification. In brief, Ub-propargylamine (Ub-PA) (Boston Biochem) was dissolved to 2 µg/µl in dimethyl sulfoxide (DMSO) and finally diluted in 1× probe buffer (100 mM NaCl, 25 mM Tris [pH 7.5]). Three molar equivalents (relative to recombinant TgOTUD3A proteins) of Ub-PA was incubated with wild type and TgOTUD3A (C229A) in a 20-µl reaction mixture for 30 min at 37°C. The reactions were stopped by addition of 2× Laemmli SDS-PAGE sample buffer, and the reaction mixtures were incubated for another 15 min at 37°C before being run on a 4 to 20% Tris-glycine gradient gel. The gel was stained with Coomassie blue to visualize binding of Ub-PA to the recombinant TgOTUD3A proteins. Duplicate reaction mixtures were run on SDS-PAGE gels and immunoblotted with mouse polyclonal TgOTUD3A antibody.
